# Characteristics of Preoperative Arteriosclerosis Evaluated by Cardio-Ankle Vascular Index in Patients with Osteoarthritis before Total Knee Arthroplasty

**DOI:** 10.3390/jcm12144685

**Published:** 2023-07-14

**Authors:** Yoshinori Ishii, Hideo Noguchi, Junko Sato, Ikuko Takahashi, Hana Ishii, Ryo Ishii, Kei Ishii, Kai Ishii, Shin-ichi Toyabe

**Affiliations:** 1 Ishii Orthopaedic & Rehabilitation Clinic, Saitama 361-0037, Japan; hid_166super@mac.com (H.N.); jun-sato@hotmail.co.jp (J.S.); itakahashi110@gmail.com (I.T.); 2 School of Plastic Surgery, Kanazawa Medical University, Ishikawa 920-0253, Japan; hanamed12@gmail.com; 3 Shinshu University Hospital, Nagano 390-8621, Japan; kmuyakyu@gmail.com; 4 Iwate Prefectural Chuo Hospital, Iwate 020-0066, Japan; kei141.0852@gmail.com; 5 Kouseiren Takaoka Hospital, Toyama 933-8555, Japan; kai.nd1209@live.com; 6 Niigata University Crisis Management Office, Niigata University Hospital, Niigata University Graduate School of Medical and Dental Sciences, Niigata 951-8520, Japan; toyabe@med.niigata-u.ac.jp

**Keywords:** osteoarthritis, arteriosclerosis, cardio-ankle vascular index, total knee arthroplasty, age, body mass index

## Abstract

Purpose: Cardiovascular disease (CVD) is a major risk factor for mortality in patients with osteoarthritis, and comorbidities increase postoperative complications after total knee arthroplasty (TKA). Arteriosclerosis plays a main role in hemodynamic dysfunction and CVD; however, arteriosclerosis has not been preoperatively evaluated before TKA using the cardio-ankle vascular index (CAVI). In this study, we evaluated the degree of preoperative arteriosclerosis using the CAVI in patients undergoing TKA, as well as its correlations with several preoperative patient factors. Methods: Arteriosclerosis was evaluated in 209 consecutive patients (251 knees) with osteoarthritis who underwent TKA at our institution between May 2011 and June 2022. The CAVI was measured in the supine position 1 day before TKA, and the correlations between the CAVI and several clinical factors were analyzed. Results: The CAVI was normal in 62 knees (25%), borderline in 71 knees (28%), and abnormal in 118 knees (47%). Univariate analysis revealed a moderate positive correlation between preoperative CAVI and age (r = 0.451, *p* < 0.001) and a weak negative correlation between preoperative CAVI and body weight (r = −0.306, *p* < 0.001) and body mass index (BMI) (r = −0.319, *p* < 0.001). Multivariate analysis showed that age (β = 0.349, *p* < 0.001) and BMI (β = −0.235, *p* < 0.001) were significantly correlated with preoperative CAVI. Conclusion: Arteriosclerosis should be carefully managed intraoperatively and postoperatively in patients with osteoarthritis undergoing TKA, particularly in older patients and patients with a low BMI.

## 1. Introduction

Patients with osteoarthritis experience walking difficulty due to swelling, pain, or stiffness in the affected joints [1]. This leads to decreased mobility, which is a cardiovascular (CV) risk factor [2]. Kendzerska et al. [2] concluded that increased attention to the management of osteoarthritis with the aim of improving mobility may lead to a reduction in CV events. Patients with osteoarthritis of the hip and knee have a higher risk of mortality than does the general population [3], and major risk factors reported by Nüesch et al. [3] include pre-existing diabetes, cancer, CV disease (CVD), and gait disturbance. The authors concluded that the management of patients with osteoarthritis and gait disturbance should focus not only on increasing physical activity but also on the effective treatment of CV risk factors and comorbidities. Arteriosclerosis (AS) plays a main role in hemodynamic dysfunction characterized by excessive pulsation, i.e., CVD [4]. Therefore, it is important to verify the progression of AS in patients with osteoarthritis. Although no studies to date have provided conclusive results, several systematic multicenter analyses have revealed correlations between AS and osteoarthritis [5,6,7]. The cardio-ankle vascular index (CAVI) is a marker of arterial stiffness based on stiffness parameter β and was developed in 2004 [8]. Measurement of the CAVI is simple and well-standardized, and its reproducibility and accuracy are acceptable [4]. Thus, the CAVI is a promising diagnostic tool for evaluating arterial stiffness [9]. In addition, a recent meta-analysis of the Asian population confirmed that the CAVI is an independent risk factor for CVD [10].

Total knee arthroplasty (TKA) is a reliable procedure for pain relief and functional improvement in patients with knee osteoarthritis [11,12,13]. Comorbidities, rather than age, are responsible for the increase in postoperative morbidity after TKA, and preoperative risk assessment should be optimized to reduce complications [14]. To the authors’ knowledge, however, the preoperative evaluation of AS (one of the comorbidities in patients undergoing TKA) using the CAVI has not been performed. The preoperative assessment of the severity of AS in patients with osteoarthritis is beneficial to verify the correlations between AS and osteoarthritis and to take measures against AS during the TKA procedure and in the early postoperative period.

Therefore, the purpose of the present study was to evaluate the CAVI in patients with osteoarthritis before TKA and to identify influential factors. The clinical significance of this study is that it will clarify the correlation between knee osteoarthritis requiring TKA and the degree of preoperative AS while identifying those patients for whom AS interventions are necessary before TKA.

## 2. Materials and Methods

This prospective study was conducted at our institute from May 2011 to June 2022. Informed consent was obtained from all patients after a discussion of the study, which included a description of the protocol and possible CAVI measurement-related complications. The institutional review board approved the study before commencement. In total, 209 consecutive patients (251 knees) undergoing TKA were investigated. The preoperative diagnosis indicating TKA was primary osteoarthritis. Patients who had undergone revision arthroplasties or previous tibial osteotomies and patients with rheumatoid arthritis were excluded.

The following preoperative factors were analyzed: sex, age, body mass index (BMI), body weight (BW), blood cholesterol level, blood triglyceride level, smoking history, diabetes mellitus, hypertension (all of which have been previously reported to affect the CAVI [15,16,17,18,19]), body height, American Society of Anesthesiologists (ASA) grade [20], Kellgren–Lawrence (KL) classification [21], Hospital for Special Surgery (HSS) knee score [22], and knee range of motion. The severity of knee osteoarthritis was radiographically scaled using the KL grading system as follows: very mild (grade I), mild (grade II), moderate (grade III), and severe (grade IV) [21]. All TKAs were evaluated using the HSS knee score [22], which is not a patient-derived score but a physician-derived score. The HSS knee score is divided into seven categories: pain, function, range of motion, muscle strength, flexion deformity, instability, and subtraction.

### 2.1. Measurement of CAVI

**Figure 1:**
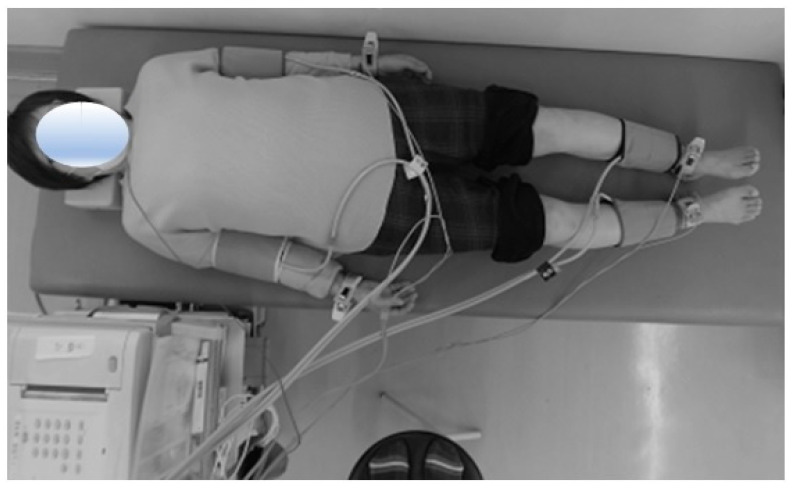
Measurement of the cardio-ankle vascular index (CAVI). First, the distance from the origin of the aorta to the ankle was measured with the patient lying in the supine position on a bed at rest. Next, cuffs used to measure blood pressure were wrapped around the right and left upper arms as well as the right and left ankle joints, and a microphone that detects heart sounds was attached to the chest. At the flip of a switch, the instrument automatically measured the pulse wave and blood pressure and calculated the CAVI. The entire measurement took about 15 min, and the test was painless.

The CAVI was measured by the standardized method using a noninvasive blood pressure-independent device (VaSera VS-1 3000; Fukuda Denshi, Tokyo, Japan) [23] at 1 day before surgery. The examination was performed in a room in which a standard temperature was maintained. In brief, the CAVI measurements were performed in the supine position. Cuffs were applied bilaterally to the upper arms and lower legs superior to the ankles. Electrocardiogram electrodes and a microphone were placed on both wrists, both ankles, and the sternum. An electrocardiogram, blood pressure, and waveforms of the brachial and ankle arteries were measured (Figure 1). The pulse wave velocity (PWV) was calculated by measuring the time between the closing sound of the aortic valve, the notch of the brachial pulse wave, and the ankle pulse wave. Using this value, the CAVI was calculated by the following equation: CAVI = 2ρ/(systolic blood pressure − diastolic blood pressure) × (ln systolic blood pressure/diastolic blood pressure) × PWV^2^, where ρ = blood viscosity. The CAVI cutoff values of 8 and 9 were proposed by the Japan Society for Vascular Failure (<8, normal; 8 to <9, borderline; and ≥9, abnormal) [24].

### 2.2. Reproducibility

To eliminate interobserver variability, all tests were performed by the same observer. Test–retest reliability was assessed using intraclass correlation coefficients, which were performed by the same observer on 30 patients at 1-month intervals. The intraclass correlation coefficient was calculated to be 0.788 (0.603–0.898).

### 2.3. Statistical Analysis

Because data for certain variables did not pass the Kolmogorov–Smirnov normality test or Shapiro–Wilk normality test, we used the non-parametric Wilcoxon rank sum test and Spearman’s rank correlation test. Univariate and multivariate analyses were performed to examine factors related to the preoperative CAVI. Spearman’s rank correlation coefficient was used to investigate the association between the preoperative CAVI and each variable. The strength of the correlation of the rank coefficients was defined as strong (0.70–1.00), moderate (0.40–0.69), or weak (0.20–0.39). The Wilcoxon rank sum test was used to determine differences in the CAVI between two groups. Multiple linear regression analysis was performed to identify variables significantly associated with the preoperative CAVI. Multiple linear regression models were constructed by entering all variables shown in Table 1, and variables significantly associated with the preoperative CAVI were selected using the stepwise selection method. In all tests, a *p* value of <0.05 was considered significant. All statistical analyses were performed using IBM SPSS Statistics version 23 (IBM Japan, Tokyo, Japan). The values are expressed as median (25th percentile, 75th percentile) (minimum–maximum).

## 3. Results

**Figure 2:**
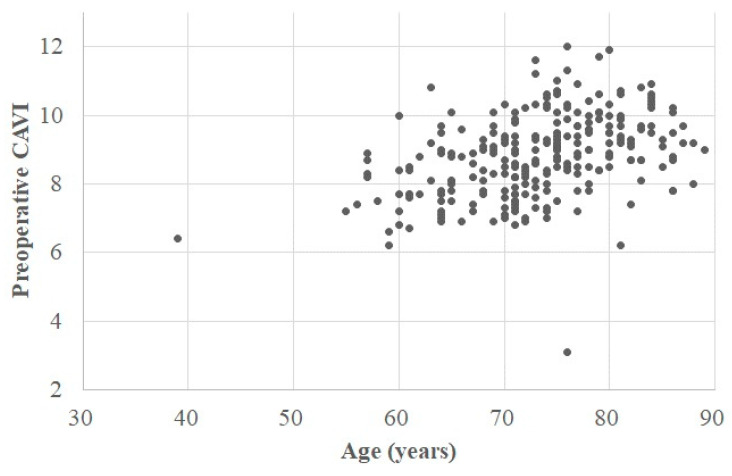
Scatterplot of preoperative CAVI and age. The horizontal axis indicates patient age, and the vertical axis indicates the preoperative CAVI. Correlation equation: CAVI = 4.064 + 0.065 × AGE. CAVI, cardio-ankle vascular index.

**Figure 3:**
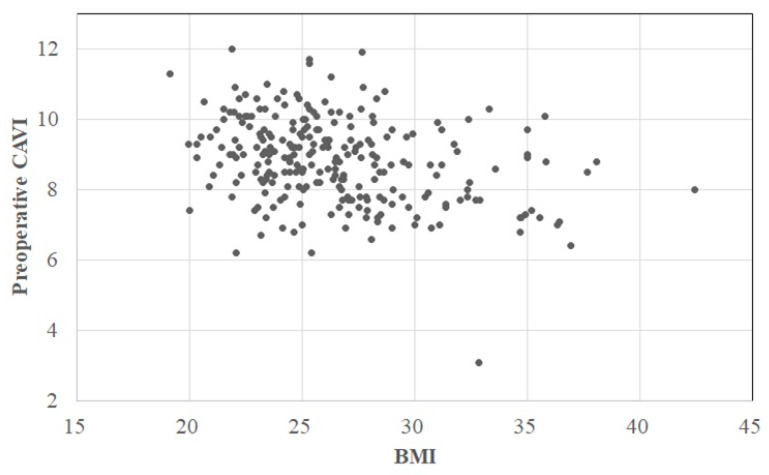
Scatterplot of preoperative CAVI and BMI. The horizontal axis indicates the BMI, and the vertical axis indicates the preoperative CAVI. Correlation equation: CAVI = 11.445 − 0.099 × BMI, CAVI, cardio-ankle vascular index; BMI, body mass index.

The patients’ clinical backgrounds are summarized in Table 1. The preoperative CAVIs in the operative and contralateral knee were 8.9 (8.0, 9.7) (3.1–12.0) and 8.9 (8.0, 9.7) (3.1–13.2), respectively. In accordance with the cutoff values of the CAVI, the CAVI was defined as normal in 62 (25%) knees, borderline in 71 (28%), and abnormal in 118 (47%) (Figure 2 and Figure 3).

According to the univariate analyses using Spearman’s correlation coefficient for continuous variables, there was a moderate positive correlation between age and the preoperative CAVI (r = 0.451, *p* < 0.001) (Figure 2) and a weak negative correlation between BW/BMI and the CAVI (r = −0.306/−0.319, *p* < 0.001/*p* < 0.001) (Table 2) (Figure 3). However, the other study variables (both continuous and discrete) showed no significant correlations (Table 2 and Table 3).

Finally, based on the multivariate analyses using multiple linear regression analysis with stepwise variable selection, the age and BMI were significantly correlated with the preoperative CAVI (β = 0.349, *p* < 0.001 and β = −0.235, *p* < 0.001, respectively) (Table 4).

## 4. Discussion

This study produced two important findings. First, we found a positive correlation of the preoperative CAVI (or AS) with age and a negative correlation of the CAVI with BMI and BW. Second, there were no correlations between AS and factors previously reported to impact AS, such as sex [15,16,17], hypertension [15,16,17], diabetes mellitus [15,16], the triglyceride level [17,18], the cholesterol level [16,17], and smoking [15,19].

Shirai et al. [23] reported that worsening of the CAVI with age occurs at a rate of 0.5 per decade in the Japanese general population according to the linear regression equation. If we calculate the CAVI using the same linear regression equation (5.43 + 0.053x age for males and CAVI = 5.34 + 0.049x age for females) in the general population, the equation performed separately for males and females in the present study would yield a CAVI of 9.5 for males because they were 76 years old and 8.9 for females because they were 73 years old. Thus, the median CAVI of 8.9 at the age of 74 years, including both males and females with end-stage osteoarthritis in this study, is comparable to that in the general population. Finally, the multivariate analysis showed that age was the strongest factor affecting AS in patients with osteoarthritis.

Another finding of this study is that the BW and BMI were negatively correlated with CAVI, suggesting that some muscle mass and fat are necessary for maintenance of the CAVI or prevention of its deterioration. Two previous studies support our results. Park et al. [25] stated that low muscle mass is independently and significantly associated with an increased CAVI and should be considered when assessing the risk of atherosclerosis in asymptomatic patients. Nagayama et al. [16] speculated that systemic accumulation of adipose tissue may itself lead to a linear reduction in arterial stiffness in non-obese and obese patients without metabolic disorders. The significant correlation of the BMI with the CAVI in the present study also suggests that proper muscle mass and moderate adipose tissue may have a positive effect on AS. Thus, the present study may suggest that the patient characteristic that warrants caution regarding AS during and immediately after TKA is a lean body habitus (low BMI) in patients of advanced age.

In the present analysis, the CAVI was not correlated with factors other than age, BW, and BMI, as previously reported [15,16,17,18,19]. This result does not mean that preoperative complications and comorbidities do not impact the CAVI, but the fact that all patients in this study had an ASA of I or II suggests that their clinical condition had little impact on the CAVI or that they were successfully treated. This is a reasonable assumption given that a preoperative ASA score of ≥3 has been reported to be an independent risk factor for serious adverse events after TKA [26]. The finding that less than half of the patients (47%) had a preoperative CAVI of ≥9.0 and were judged abnormal [23] seems to corroborate the conclusion that preoperative comorbidities were not severe in this study.

Finally, there was no correlation between the preoperative CAVI and the degree of osteoarthritis by the KL classification [21] or HSS knee score [22], suggesting that increased pain and decreased walking ability in association with the severity of osteoarthritis may not play a major role in the progression of the CAVI or AS. However, considering previous reports of higher all-cause mortality in patients with osteoarthritis than in the general population [3] and reports that the severity of osteoarthritis-related disability is associated with significantly increased all-cause mortality and serious CVD events [27] (also demonstrating the association between osteoarthritis and comorbidities, including AS), osteoarthritis may play a supporting role in amplifying AS-aggravating factors such as diabetes, hypertension, and hyperlipidemia.

This study had three limitations. First, this study was conducted at a single institution; thus, the distribution of the patients was skewed, with a disproportionate number of males and females and only mild comorbidity in patients with ASA classifications of I and II. Future studies should analyze patients with various backgrounds at multiple centers. Second, the analysis was limited to Japanese patients. Interestingly, several studies have suggested differences in the mean CAVI among countries [28,29,30]. Therefore, multinational studies should be performed to verify the validity of our results. Third, the HSS clinical scores, including activity assessment [22], were evaluated prior to TKA surgery, but specific measures of activity, such as the number of steps, were not evaluated. Specific step counts are generally confirmed using pedometers. Despite these limitations, the main strength of this study is that it is the first report of AS evaluation using the CAVI with a focus on patients with osteoarthritis. Furthermore, not only do the results of this study clarify the patient population that is likely to require AS countermeasures intraoperatively and immediately postoperatively, but the results also make it possible to confirm the spillover effects of TKA on AS if CAVI trends after TKA surgery are observed over the middle to long term.

## 5. Conclusions

The results of this study suggest the following:

1. The patient characteristics that warrant special attention to AS intraoperatively and immediately postoperatively are a lean body habitus (low BMI) and advanced age.
2. Future studies based on the accumulation of preoperative CAVI data in patients with osteoarthritis who have various backgrounds, including patients with an ASA score of ≥III, are essential to more practically evaluate the impact of end-stage osteoarthritis on AS.

## Figures and Tables

**Table 1:** Patients’ backgrounds.

Variables (Patients/Knees)	209/251
Sex (male vs. female)	42/209
Body height (cm)	150 (146, 155)
Body weight (kg)	59 (53, 67)
Body mass index (kg/m^2^)	26 (24, 28)
Age (years)	74 (69, 79), M; 76 (70, 81), F; 73 (69, 78)
Smoking history (yes/no)	12/239
Diabetes mellitus (yes/no)	35/216
Hypertension (yes/no)	164/87
Preop. blood cholesterol level (mg/dL)	205 (185, 234)
Preop. blood triglyceride level (mg/dL)	132 (101, 175)
Knee flexion (Preop) (°)	115 (100, 125)
Knee extension (Preop) (°)	−10.0 (−15, −5)
Knee range of motion (Preop) (°)	100 (90, 120)
HSS score [22]	45 (37, 52)
Kellgren–Laurence classification [21]	I 0, II 0, III 10, IV 241
ASA grade [20]	I 34, II 217

Data are presented as n or median (25th percentile, 75th percentile). M, male; F, female; Preop, preoperative; HSS, Hospital for Special Surgery; ASA, American Society of Anesthesiologists.

**Table 2:** Correlations between preoperative CAVI and study variables by Spearman’s rank correlation coefficient.

Variables	r	*p*
Pre CAVI (contra-lateral)	0.901	*p* < 0.001
**Age**	**0.451**	***p*** < **0.001**
Body height	−0.049	0.442
**Body weight**	**−0.307**	***p*** < **0.001**
**Body mass index**	**−0.322**	***p*** < **0.001**
HSS score [22]	−0.089	*p* = 0.160
Flexion	0.069	0.275
Extension	−0.096	0.130
ROM	0.024	0.700
Cholesterol	0.025	0.695
Triglyceride	0.044	0.484

Values in bold indicate statistically significant values. CAVI, cardio-ankle vascular index; HSS, Hospital for Special Surgery; ROM, range of motion.

**Table 3:** Comparison of preoperative CAVI in discrete study variables by Wilcoxon rank sum test.

Variables Knees (Patients)	Median (Interquartile) Range		*p*
Sex: male/female 42 (36) (17%)/209 (173) (83%)	Male; 9.1 (8.4, 9.7) 6.4–11.9	Female; 8.8 (7.8, 9.6) 3.1–12.0	0.223
KL [21]: III 10 (4%), IV 241 (96%)	III; 9.1 (8.0, 9.4) 6.9–10.6	IV; 8.8 (8.0, 9.7) 3.1–12.0	0.950
ASA [20]: I 34 (14%), II 217 (86%)	I; 8.9 (7.8, 9.9) 6.2–11.6	II; 8.9 (8.0, 9.6) 3.1–12	0.691
Smoking history: yes 12 (5%)	Yes; 8.8 (8.5, 9.3) 6.7–10.4	No; 8.9 (8.0, 9.7) 3.1–12.0	0.987
Hypertension: yes 164 (65%)	Yes; 9.0 (8.1, 9.7) 3.1–12.0	No; 8.7 (7.7, 9.6) 6.2–11.7	0.078
Diabetes mellitus: yes 35 (14%)	Yes; 8.9 (8.0, 9.5) 7.0–11.6	No; 8.8 (8.0, 9.7) 3.1–12.0	0.962

Data are presented as n or median (25th percentile, 75th percentile) (minimum–maximum). CAVI, cardio-ankle vascular index; KL, Kellgren–Lawrence classification; ASA, American Society of Anesthesiologists.

**Table 4:** Results of multiple regression analysis using stepwise variable selection.

	B	S.E.	β	Sig.	95% CI	
(Constant)	6.699	0.920		<0.001	4.887	8.512
Age	0.055	0.009	0.349	<0.001	0.035	0.073
BMI	−0.072	0.015	−0.235	<0.001	−0.107	−0.037

BMI, body mass index; S.E., standard error; Sig., significance; CI, confidence interval.

## Data Availability

The datasets used and/or analyzed during the current study are available from the corresponding author on reasonable request.
